# Examining the impact perceptual learning artificial-intelligence-based on the incidence of paresthesia when performing the ultrasound-guided popliteal sciatic block: simulation-based randomized study

**DOI:** 10.1186/s12871-022-01937-6

**Published:** 2022-12-16

**Authors:** Nan Cai, Geng Wang, Li Xu, Yan Zhou, Hao Chong, Yaoping Zhao, Jingxian Wang, Wenjia Yan, Bo Zhang, Nan Liu

**Affiliations:** 1 Department of Anesthesiology, Beijing Jishuitan Hospital, Beijing, 100000 China; 2 Beijing AMIT Healthcare, Beijing, 100000 China

**Keywords:** Artificial intelligence, Perceptual learning, Ultrasound guidance, Nerve block, Sciatic nerve block via the popliteal approach, Teaching, Simulation-based randomized study

## Abstract

**Objective:**

To explore the impact of artificial-intelligence perceptual learning when performing the ultrasound-guided popliteal sciatic block.

**Methods:**

This simulation-based randomized study enrolled residents who underwent ultrasound-guided sciatic nerve block training at the Department of Anesthesiology of Beijing Jishuitan Hospital between January 2022 and February 2022. Residents were randomly divided into a traditional teaching group and an AI teaching group. All residents attended the same nerve block theory courses, while those in the AI teaching group participated in training course using an AI-assisted nerve identification system based on a convolutional neural network instead of traditional training.

**Results:**

A total of 40 residents were included. The complication rates of paresthesia during puncture in the first month of clinical sciatic nerve block practice after training were significantly lower in the AI teaching group than in the traditional teaching group [11 (4.12%) vs. 36 (14.06%), *P* = 0.000093]. The rates of paresthesia/pain during injection were significantly lower in the AI teaching group than in the traditional teaching group [6 (2.25%) vs. 17 (6.64%), *P* = 0.025]. The Assessment Checklist for Ultrasound-Guided Regional Anesthesia (32 ± 3.8 vs. 29.4 ± 3.9, *P* = 0.001) and nerve block self-rating scores (7.53 ± 1.62 vs. 6.49 ± 1.85, *P* < 0.001) were significantly higher in the AI teaching group than in the traditional teaching group. There were no significant differences in the remaining indicators.

**Conclusion:**

The inclusion of an AI-assisted nerve identification system based on convolutional neural network as part of the training program for ultrasound-guided sciatic nerve block via the popliteal approach may reduce the incidence of nerve paresthesia and this might be related to improved perceptual learning.

**Clinical trial:**

CHiCTR2200055115, registered on 1/ January /2022.

## Introduction

Nerve block is widely used to reduce perioperative pain in patients. Successful nerve block requires effective distribution of the local anesthetic around the nerve. Ultrasound-guidance can substantially improve the quality of the nerve block [1–3]. The successful implementation of ultrasound-guided nerve blocks depends on the experience and skill of the operator [4, 5].

The initial challenge is to interpret the ultrasound image and identify the relevant anatomical structures form beginning [6]. Although it is recognized that ultrasound-guided regional anesthesia requires a good knowledge of anatomy, the issue of image interpretation has received relatively little attention [7]. Gaps in the anesthesiologist’s knowledge of anatomy and inter-individual anatomical variations can result in the suboptimal interpretation of ultrasound images and difficulties in identifying anatomical structures [8–10]. Currently, most peripheral nerve blocks are performed by a limited number of specialists, reflecting the challenges and complexities of these procedures [11].

Perceptual learning can be defined as “an increase in the ability to extract information from the environment, as a result of experience and practice with stimulation coming from it [12].” Perceptual learning interventions increased the short- and long-term diagnostic performance of trainees by efficiently teaching pattern recognition and categorization. It can be used at different stages of training in several medical fields to improve diagnostic speed and accuracy in academic tests with possible acceleration of the development of professional expertise [13].

Artificial intelligence (AI), is a branch of computer science that enables smart machines to solve problems and perform tasks. The use of AI in medicine is increasing [14]. There is particular interest in the application of AI to medical imaging [15], for example to improve the diagnostic performance of imaging modalities [16, 17]. Since ultrasound-guided nerve block relies on imaging, AI potentially could be used to enhance image optimization and interpretation in real-time, which in turn would help physicians to identify the target nerve and avoid complications.

From the perspective of learning, perceptual training and machine learning were very similar. We present a large number of training images for human or machine, one-by-one, attempts to classify each image according to a preset criterion and is informed whether they were correct. Based on this feedback, learning can be increased by increasing the number of training images [18].

It was hypothesized that an AI-assisted identification system would help to improve the perceptual learning of residents for the ultrasound-guided nerve block and reduce the rate of complications during the initial period of their clinical work. Therefore, the present study aimed to examining the impact perceptual learning artificial-intelligence-based on the incidence of paresthesia when performing the ultrasound-guided popliteal sciatic block.

## Methods

### Study design and participants

This study enrolled residents receiving standardized training or a refresher program in the Department of Anesthesiology of Beijing Jishuitan Hospital between January 2022 and February 2022. The inclusion criteria were as follows: 1) age ≥ 18 years; 2) less than five cases of ultrasound-guided sciatic nerve block was performed before. The exclusion criteria were: 1) hearing or visual impairment; and 2) unable to complete the full-term study. This study was approved by the ethics committee of Beijing Jishuitan Hospital (No. 202106–41) and was registered at the Chinese clinical trial registry (CHiCTR2200055115). Written informed consents were obtained from all participants.

### Study intervention and procedures

**Fig. 1:**
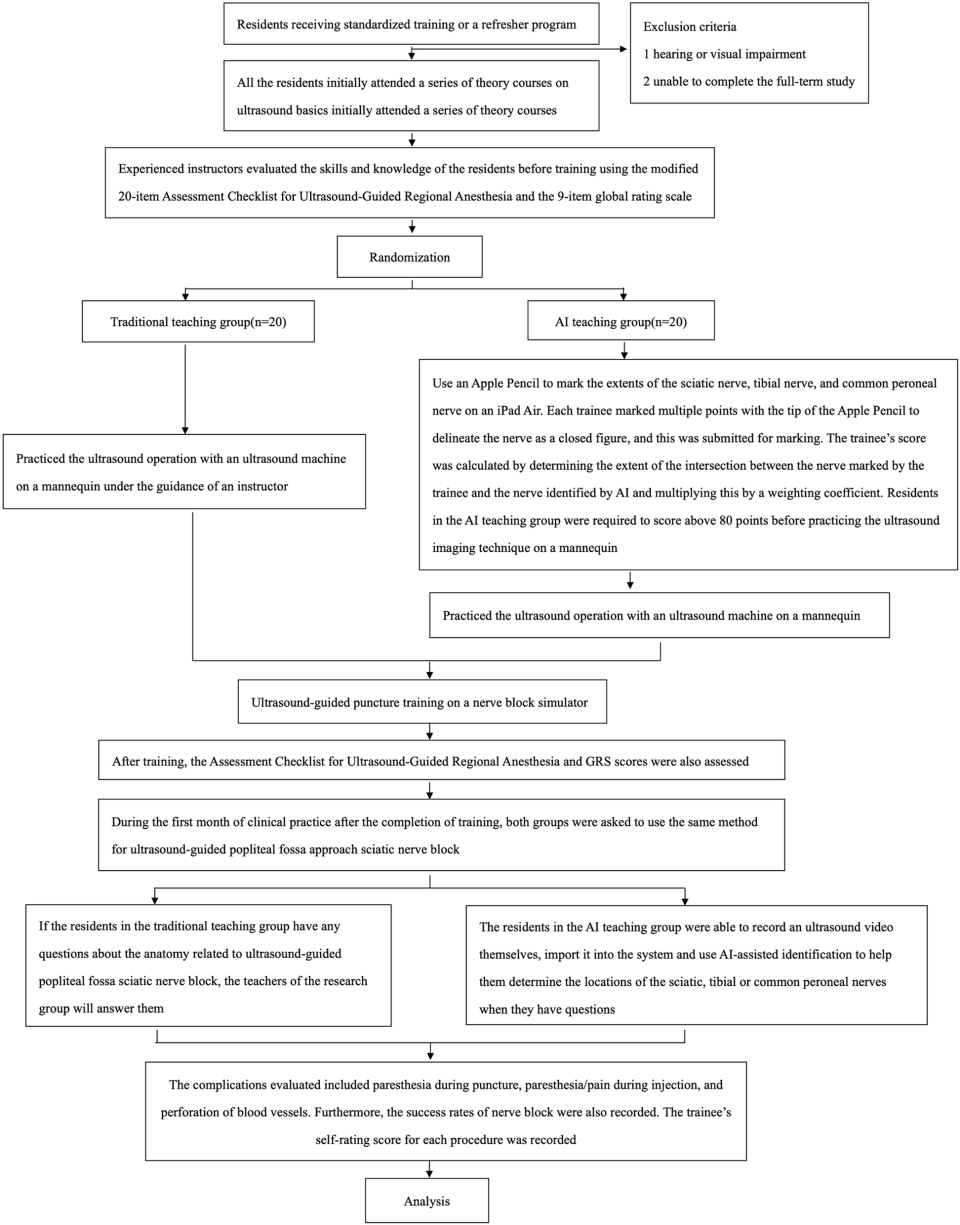
Study flow diagram

The research process can be seen in Fig. 1.

**Fig. 2:**
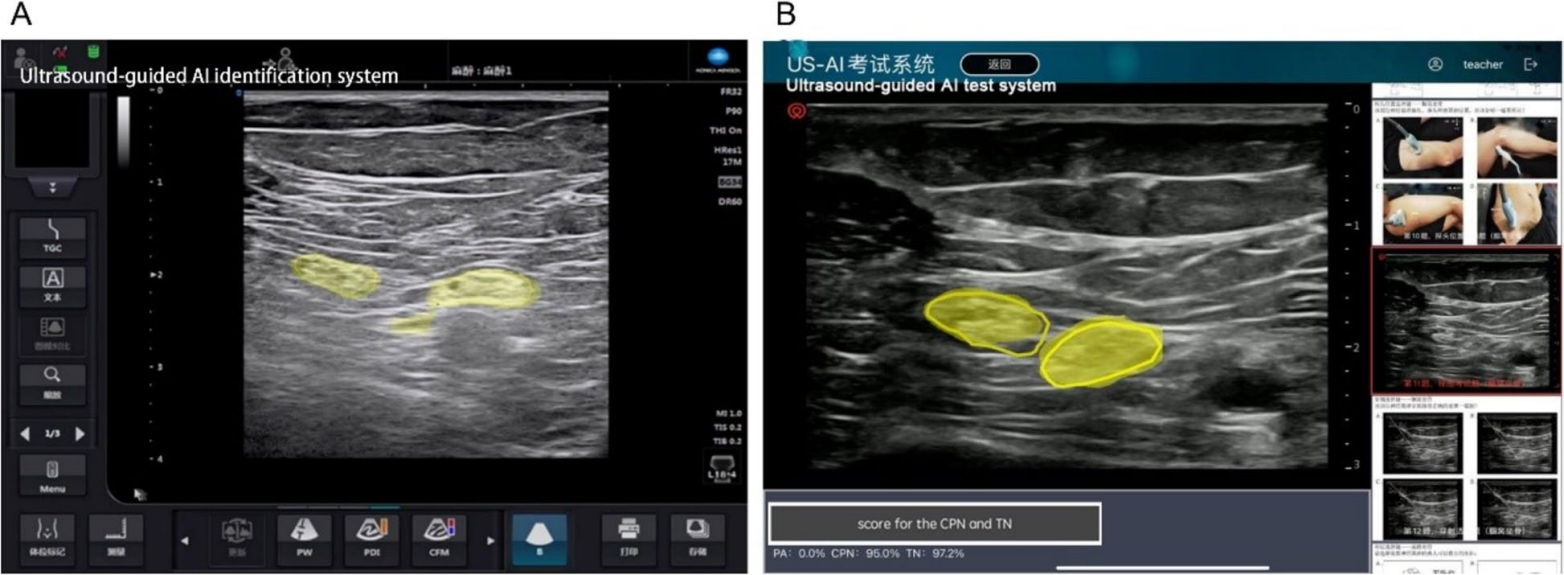
Artificial intelligence-assisted identification system for ultrasound-guided sciatic nerve block training. **A** AI-assisted identification system used to train residents to perform ultrasound-guided sciatic nerve block via the popliteal approach. The yellow areas represent the TN and CPN. **B** AI-assisted test system to give feedback to residents. The test score was calculated by determining the extent of the intersection between the nerve area marked by the trainee (yellow area) and that identified by AI (area inside the yellow line)

The AI-assisted identification system for ultrasound-guided nerve block was established based on convolutional neural networks and developed by acquiring videos of ultrasound-guided sciatic nerve block via the popliteal fossa. Specific structures (mainly the sciatic, tibial, and common peroneal nerves) in each frame were annotated by decomposing each captured video into multiple frames. The labeled frames which completed by 15 experienced doctors were used to train a machine-learning algorithm that utilized deep learning to establish associations between the labels and the underlying structures. This allowed the extent of the target nerve to be displayed in real-time on the ultrasound scan of the corresponding site (Fig. 2A). A total of 1721 images were used to train and validate the AI-assisted identification system (training set: validation set: test set = 7:2:1). To ensure the validity, 6 experts of ultrasound guide nerve block were recruited to assess the AI-assisted identification system.

All the residents initially attended a series of theory courses on ultrasound basics initially attended a series of theory courses on ultrasound basics, ultrasound use and image optimization, sciatic nerve anatomy, and the ultrasound-guided sciatic nerve block technique. The duration of each course was 30 minutes, and all residents were taught by the same experienced physicians. Randomization will be based on computer-generated allocation, and random numbers will be concealed in opaque envelopes. After the theory courses, the envelope corresponding to a number in the randomization table will be opened, and the residents will be randomly divided into a traditional teaching group and an AI teaching group at a 1:1 allocation ratio according to random number. After the theory classes had been completed, the residents in the traditional teaching group practiced the ultrasound operation with an ultrasound machine (KONICA MINOLTA, SONIMAGE HS1) on a mannequin under the guidance of an instructor. In the AI teaching group, residents were asked to use an Apple Pencil to mark the extents of the sciatic nerve, tibial nerve, and common peroneal nerve on an iPad Air (Apple, USA), which was part of the AI-based teaching system. Each trainee marked multiple points with the tip of the Apple Pencil to delineate the nerve as a closed figure, and this was submitted for marking. The trainee’s score was calculated by determining the extent of the intersection between the nerve marked by the trainee and the nerve identified by AI and multiplying this by a weighting coefficient (Fig. 2B). Residents in the AI teaching group were required to score above 80 points before practicing the ultrasound imaging technique on a mannequin. (Total duration for each group in this course was 1 h) After the two groups of residents had completed the mannequin-based practice, they underwent ultrasound-guided puncture training on a nerve block simulator (MiniSim Popliteal Trainer, Valkyrie).

During the first month of clinical practice after the completion of training, both groups were asked to use the same method for ultrasound-guided popliteal fossa approach sciatic nerve block. (The ultrasound probe was placed transversely across the popliteal fossa at the popliteal crease. After confirming the popliteal artery, vein, and nerve, the probe was moved proximally to find the bifurcation of the sciatic nerve. The neural bifurcation was identified as the point where both branches are contiguous and display a bilobular pattern. Using an in-plane technique, needle was advanced from lateral. After identifying the paraneural sheath, the needle was advanced within the paraneural sheath. At this point, a previously determined volume of ropivacaine 0.5% was injected slowly after negative blood aspiration).

If the residents in the traditional teaching group have any questions about the anatomy related to ultrasound-guided popliteal fossa sciatic nerve block, the teachers of the research group will answer them. The residents in the AI teaching group were able to record an ultrasound video themselves, import it into the system and use AI-assisted identification to help them determine the locations of the sciatic, tibial, or common peroneal nerves when they have questions.

### Outcomes

Before training, all residents were asked to complete a survey to record their age, gender, years of experience working in anesthesia, the number of nerve block procedures completed previously, previous training in ultrasound-guided nerve block, the number of nerve blocks performed monthly in their place of work, the type of ultrasound-guided nerve block training received previously, and their confidence to perform ultrasound-guided sciatic nerve block via the popliteal fossa independently. Experienced instructors evaluated the skills and knowledge of the residents before training using the modified 20-item Assessment Checklist for Ultrasound-Guided Regional Anesthesia and the 9-item global rating scale (GRS) [19]. The Assessment Checklist for Ultrasound-Guided Regional Anesthesia consists of specific items scored on a scale of 0–2 (0, not completed; 1, completed with prompting; 2, completed well without prompting) and has a maximal score of 40 points. The GRS is a 5-point scale containing nine items related to ultrasound-guided nerve block preparation, the patient, and technique, and the maximal score is 45 points. A higher score indicates better performance on both assessment scales.

After training, the Assessment Checklist for Ultrasound-Guided Regional Anesthesia and GRS scores were also assessed. Complications related to ultrasound-guided sciatic nerve block via the popliteal fossa were evaluated within the first month of clinical practice after training. The complications evaluated included paresthesia during puncture, paresthesia/pain during injection, and perforation of blood vessels. Furthermore, the success rates of nerve block were also recorded. The trainee’s self-rating score for each procedure was recorded on a scale of 0–10, with 10 being the highest. Self-rating score questionnaires with incomplete information were excluded.

The main outcome were the complication rates of paresthesia during puncture in the first month after training. The secondary outcomes were the complication rates included paresthesia/pain during injection, perforation of blood vessels, the success rates of nerve block. And the self-rating scores and the Assessment Checklist for Ultrasound-Guided Regional Anesthesia and GRS scores after training.

### Statistical analysis

SPSS 22.0 (IBM, Armonk, NY, USA) was used for data analysis. Data for complication rate of paresthesia during puncture, paresthesia or pain during injection, penetration of a blood vessel, successful rate of nerve block were compared between the two groups using chi-squared test or Fisher’s exact test and presented as cases (percentage). Data for self-rating score were assessed using independent sample t-tests and presented as means ± standard deviation. Post-training Assessment Checklist for Ultrasound-Guided Regional Anesthesia and GRS scores were compared between the two groups using covariate analysis of variance (ANCOVA) with the baseline score as the covariate. Two-sided *P* < 0.05 was considered to indicate a statistically significant difference. Data for the age of the residents were using Hodges-Lehmann estimation to calculate the 95% CI of the median differences observed between the two groups and presented as medians (interquartile range). The other baseline characteristics of the residents were using Clopper-Pearson exact method to calculate 95% CI of the odds ratio between the two groups and presented as cases (percentage).

### Sample size estimation

The sample size was determined by a power analysis based on the complication rate of paresthesia during puncture in our previous pilot study. The complication rate of paresthesia during puncture for the traditional teaching group was about 14% and we expect that the AI teaching group will exhibit about 50% reduced. Based on these data we found that 217 procedures per group will provide a difference at a significance level of 0.05. In addition, every resident usually played 15 ultrasound-guided sciatic nerve blocks during a month (at least 15 person). Comprehensive considering a possible dropout rate of 10%, 20 residents will be included per group.

## Results

Forty residents were enrolled in this study, all of whom completed the training programs. The AI ​​teaching group included 20 residents (9 males) aged 34(32–36) years old, and the traditional teaching group included 20 residents (10 males) aged 33(25–36) years old. Thirteen residents in the traditional teaching group and 15 residents in the AI ​​teaching group had more than 5 years of experience in anesthesiology [65% vs. 75%, 1.65 (0.41, 6.33); Table 1]. Two residents in the traditional teaching group and 6 residents in the AI teaching group had performed more than 20 other nerve block procedures [10% vs. 30%, 3.86(0.67, 22.11); Table 1]. Three residents in the traditional teaching group and 2 in the AI ​​teaching group were confident about independently performing ultrasound-guided sciatic nerve block via the popliteal fossa [15% vs. 10%, 1.59 (0.24, 10.7); Table 1]. There were no significant differences between groups in age, gender, work experience in anesthesiology, and indicators related to nerve block experience and training (Table 1).

**Table 1:** Baseline characteristics of the residents

Characteristics	Traditional teaching group (*n* = 20)	AI teaching group (*n* = 20)	OR 95% CI	Estimated Difference (95% CI)
Age, years	33 (25–36)	34 (32–36)	–	-1 (−6, 2)
Male, n (%)	10 (50%)	9 (45%)	1.22 (0.35, 4.23)	
Anesthesiology experience > 5 years, n (%)	13 (65%)	15 (75%)	1.65 (0.41, 6.33)	
Nerve block procedures completed previously > 20 cases, n (%)	2 (10%)	6 (30%)	3.86 (0.67, 22.11)	
Nerve block procedures performed monthly > 10 cases, n (%)	3 (15%)	4 (20%)	1.42 (0.27, 7.34)	
Previous training in ultrasound-guided nerve block, n (%)	7 (35%)	9 (45%)	0.66 (0.18, 2.35)	
Previous training type of ultrasound-guided nerve block training, *n* (%)				
None	13 (65%)	11 (55%)	1.52 (0.43, 5.43)	
Courses	5 (25%)	7 (35%)	0.62 (0.16, 2.43)	
Learning sessions	1 (5%)	1 (5%)	1.00 (0.06, 17.18)	
Department rotation trainings	1 (5%)	1 (5%)	1.00 (0.06, 17.18)	
Had confidence to complete procedure independently	3 (15%)	2 (10%)	1.59 (0.24, 10.7)	

The residents performed 552 ultrasound-guided sciatic nerve block procedures via the popliteal fossa during the first month after training (273 procedures for the traditional teaching group and 279 procedures for the AI ​​teaching group). Seventeen incomplete self-rating score questionnaires were excluded for the traditional teaching group, and 23 were excluded for the AI ​​teaching group. Compared with the traditional teaching group, residents in the AI teaching group had a significantly lower complication rate of paresthesia during puncture [11 (4.12%) vs. 36 (14.06%), *P* = 0.000093; Table 2].

**Table 2:** Outcomes in clinical practice and the assessment checklist for ultrasound-guided regional anesthesia and GRS

	Traditional teaching group	AI teaching group	***P*** value
**Outcomes in clinical practice**	(*n* = 256)	(*n* = 267)	
**Complications,** ***n*** **(%)**			
**Paresthesia during puncture**	36 (14.06%)	11 (4.12%)	< 0.001
**Paresthesia or pain during injection**	17 (6.64%)	6 (2.25%)	0.025
**Penetration of a blood vessel**	11 (4.30%)	6 (2.25%)	0.186
**Successful rate of nerve block,** ***n*** **(%)**	226 (88.3%)	239 (89.5%)	0.68
**Self-rating score**	6.49 ± 1.85	7.53 ± 1.62	< 0.001
**Scales**	**(*n*** **= 20)**	**(*n*** **= 20)**	
**Assessment checklist for ultrasound-guided regional anesthesia**			
**before training**	19.6 ± 5.6	18.5 ± 4.5	0.496
**after training**	29.4 ± 3.9	32 ± 3.8	0.001
**GRS**			
**before training**	26.4 ± 5.6	24.0 ± 4.3	0.137
**after training**	35.2 ± 4.7	33.4 ± 5.0	0.752

*Abbreviation*: *GRS* the 9-item global rating scale

Compared with the traditional teaching group, residents in the AI ​​teaching group had a significantly lower complication rate of paresthesia/pain during injection [6 (2.25%) vs. 17 (6.64%), *P* = 0.025; Table 2]. There were no significant differences in the complication rate of blood vessel perforation between the two groups [6 (2.25%) vs. 11 (4.30%), *P* = 0.186; Table 2]. There were no significant differences in the success rate of nerve block between the two groups [239 (89.5%) vs. 226 (88.3%), *P* = 0.68; Table 2). Therefore, the final analysis of self-rating scores included 256 valid questionnaires for the traditional teaching group and 267 for the AI ​​teaching group. Notably, the self-rating score after training was significantly higher in the AI ​​teaching group than in the traditional teaching group (7.53 ± 1.62 vs. 6.49 ± 1.85, *P* < 0.001; Table 2).

There was no significant difference in the Assessment Checklist for Ultrasound-Guided Regional Anesthesia or GRS score between the two groups before training (Table 2). Residents in both groups exhibited increases in the Assessment Checklist and GRS scores after training. The Assessment Checklist score after training was significantly higher in the AI ​​teaching group than in the traditional teaching group (32 ± 3.8 vs. 29.4 ± 3.9, *P* = 0.001; Table 2), but there was no significant difference between the two groups in the post-training GRS scale score (33.4 ± 5.0 vs. 35.2 ± 4.7).

## Discussion

A notable finding of the present study was that the rates of paresthesia during puncture and paresthesia/pain during injection were significantly lower in the AI ​​teaching group than in the traditional teaching group. After training, the Assessment Checklist for Ultrasound-Guided Regional Anesthesia and the self-rating scores were higher for the AI ​​teaching group than for the traditional teaching group. These findings suggest that using an AI-assisted nerve identification system during training for ultrasound-guided sciatic nerve block via the popliteal fossa might improve the perception teaching which is possible to help the residents to learn the anatomical knowledge related during the training and first month of clinical practice.

Learning the skills needed to perform ultrasound-guided nerve blocks can be challenging. The experience of performing ultrasound-guided nerve blocks is usually acquired ad hoc, which can result in inconsistencies in training between individual residents due to varying intervals between each learning experience and the use of different training methods. Worm et al. concluded that ultrasound-guided regional anesthesia education that focused on static ultrasound images is not sufficient, and that ultrasound videos and graphic enhancement techniques can help trainees to learn how to identify nerves using ultrasonography [20]. Wegener et al. found that a group of novice participants failed to identify more than half of the anatomical structures during ultrasonography after one basic training session, while another group of participants who received additional instructions failed to identify a third of the structures [21]. The low identification scores of these trainees suggest that the teaching of ultrasound-guided nerve blocks requires better training methods and equipment [21].

Romito, et al. developed a perceptual and adaptive learning modules (PALMs) present an alternative route for training transesophageal echocardiography (TOE) interpretation. They found that TOE PALM should be used as a tool for teaching TOE to novices. Perceptual learning instruments present a valuable adjunct to traditional medical training practices [22]. In addition there is evidence that assisted learning systems can supplement clinical teaching and facilitate the reinforcement of skills and concepts in inexperienced trainees. Simply and repeatedly emphasizing the anatomical features in ultrasound images can shorten the time needed for trainees to learn and master the relevant knowledge [23]. The present study explored the use of AI for perceptual learning that focused on developing the ability of the trainee to identify basic anatomical structures. Comparisons of the nerve courses traced by hand by the trainee with those detected by the AI identification system allowed the skill of the trainee to be evaluated and improved over the course of several sessions, thereby ensuring that the trainee reached a predefined level of competence before progressing to the next stages of training. The scores of Assessment Checklist for Ultrasound-Guided Regional Anesthesia after training were higher for the AI ​​teaching group than for the traditional teaching group, indicating that the residents in the AI ​​teaching group had learned at a faster rate due to a better ability to interpret ultrasound images and identify anatomical structures. There was no significant difference between the two groups in the post-training GRS scale score. This might because that the GRS scale contains more nontechnical items than the Assessment Checklist for Ultrasound-Guided Regional Anesthesia. These items include preparation and patient care, which cannot be enhanced using AI for perceptual learning. Although, there were no significant differences in the successful rate of nerve block between the two groups but the rates of neurological paresthesia during puncture and paresthesia/pain during injection were significantly lower in the AI teaching group than in the traditional teaching group. It reveals that the enhanced perceptual teaching through artificial intelligence can enable the residents to have good anatomy knowledge in short-term and long-term clinical practice, which is beneficial to reduce the complications of clinical operation and enhance the implying that the residents in the AI ​​teaching group had greater confidence in carrying out the nerve block procedure. There was no difference in the rate of vascular puncture between the two groups of residents, which may be because the AI system did not contain the identification practice of the popliteal artery and popliteal vein.

Several previous studies have evaluated the use of AI-based systems in ultrasound-guided regional anesthesia. Smistad et al. investigated the potential use of a deep convolutional neural network for ultrasound-guided axillary nerve block procedures and demonstrated that their system could identify the musculocutaneous, median, ulnar, and radial nerves as well as blood vessels in ultrasound images [24]. Liu et al. showed that a convolutional neural network improved the accuracy of ultrasound images and shortened the time required for the administration of regional anesthesia in patients with a scapular fracture [25]. Gungor et al. reported that an AI-based system helped inexperienced anesthesiologists to interpret anatomical structures in real-time during ultrasound-guided interscalene, supraclavicular, infraclavicular and transversus abdominis plane blocks [26]. Furthermore, Bowness et al. described the use of an AI device to improve the interpretation of ultrasound scans across nine peripheral nerve block regions by non-expert anesthesiologists [27]. However, to our knowledge, the present study is the first to use an AI-based system to help train residents to perform ultrasound-guided sciatic nerve block via the popliteal approach.

This study has some limitations. First, the ultrasound anatomy of the popliteal fossa approach sciatic nerve block is easily identifiable, which is more suitable for beginners or residents who have just contact with ultrasound-guided nerve block. AI-based nerve Identification would be more relevant in other more difficult nerve blocks for experienced students. Second, recognition of anatomy is only one aspect of ultrasound guide nerve block and there are more important elements. Our study does not focus on the tracking of needle entry trajectory and the teaching of needle tip display optimization, which is also the reason for the high incidence of complications such as nerve paresthesia in this study. Further research is needed on how to enhance the students’ ability to recognize the blocking needle through artificial intelligence. Third, the number of nerve block procedures performed during the first month of clinical practice varied between the residents. The inter-individual differences need to be considered in further studies. Finally, artificial intelligence fails to make top-down associations and is lack of humanistic care and logical reasoning advocated in the medical process. The human brain is still far more advanced than artificial intelligence.

## Conclusion

Based on the results of this study, the inclusion of an AI-assisted nerve identification system based on convolutional neural network as part of the training program for ultrasound-guided sciatic nerve block via the popliteal approach may reduce the incidence of nerve paresthesia and this might be related to improved perceptual learning. Further development and optimization of the AI system may help to bring a better learning experience to residents and a higher-quality medical service to patients.

## Data Availability

All data generated or analyzed during this study are included in this published article.

## Abbreviations

AI: Artificial intelligence
CPN: Common peroneal nerve
TN: Tibial nerve
